# Keep an Eye on AI: How Artificial Intelligence Is Redefining Patient Education in Dermatology

**DOI:** 10.1111/jocd.70225

**Published:** 2025-05-03

**Authors:** Diala Haykal

**Affiliations:** ^1^ Centre Laser Palaiseau, Private Practice Palaiseau France

**Keywords:** artificial intelligence (AI), dermatology, digital health, patient education, personalized care

Dear Editor,

The field of dermatology has been at the forefront of adopting artificial intelligence (AI) in clinical diagnostics, image recognition, and workflow optimization [1]. However, there is a growing and equally critical dimension of AI application that deserves closer attention, namely its role in patient education [2]. As we continue to witness a digital transformation in cosmetic and medical dermatology, AI presents a unique opportunity to empower patients with personalized, accessible, and engaging knowledge about their skin health.

In clinical practice, one of the persistent challenges lies in ensuring patients leave the consultation with a clear understanding of their condition, treatment plan, and the behaviors necessary to support successful outcomes [3]. Despite the best efforts of clinicians, traditional methods such as verbal explanations, printed brochures, or static websites often fall short. Patients forget key information, misunderstand instructions, or fail to adhere to complex regimens, particularly in chronic conditions like acne, melasma, rosacea, and atopic dermatitis, which require sustained engagement. AI can help bridge this gap. With the integration of machine learning algorithms, natural language processing, and computer vision, AI is capable of delivering hyper‐personalized educational content that adapts to a patient's skin type, language, cultural context, and health literacy level. AI‐powered platforms can explain the pathophysiology of skin conditions through interactive 3D models, simulate the effects of treatment over time, and answer follow‐up questions through virtual assistants that are available 24/7 [4]. These tools go far beyond the static nature of traditional patient education by providing dynamic, ongoing learning experiences. For instance, an AI‐enabled skin app might guide a patient with melasma through a visualization of pigment pathways, offer tailored sun protection tips based on their location and UV index, and send reminders to apply topical treatments. Patients with post‐procedural needs, such as after laser resurfacing or injectables, can receive AI‐curated aftercare instructions that evolve in real time, based on user feedback and healing progression. This just‐in‐time education can drastically reduce anxiety, improve adherence, and support outcomes.

Moreover, AI systems can analyze patient behavior and predict those who are at risk of disengagement or non‐compliance. Predictive analytics, when integrated into digital platforms or electronic health records, could prompt dermatologists or nurse educators to intervene early with supportive educational outreach. This is particularly valuable in cosmetic dermatology, where managing patient expectations is as important as the clinical result itself. We must, however, keep an eye on AI, not only for what it can do, but for how it is built and whom it serves. Dermatology has a well‐documented history of underrepresentation of skin of color in medical training, research, and datasets. As AI systems are trained on available data, they can unintentionally perpetuate these disparities unless diversity is intentionally prioritized. Patient education tools must reflect a broad spectrum of skin tones, cultural norms, and linguistic needs to avoid widening the gap in health literacy and outcomes.

This underrepresentation is particularly concerning for conditions like atopic dermatitis and psoriasis, which often present differently in skin of color and are more prone to misdiagnosis [5]. Several studies have highlighted the clinical variations in dermatoses across skin tones, underscoring the need for more inclusive training data in AI systems. AI‐based educational tools must therefore integrate culturally adapted content and authentic image sets to avoid perpetuating these diagnostic disparities. Furthermore, delayed recognition of serious malignancies like melanoma or angiosarcoma in patients with darker skin can lead to life‐threatening outcomes. Similarly, misdiagnosing benign dermatoses as malignant can lead to inappropriate treatments, patient anxiety, and alterations in skin appearance that further complicate accurate diagnosis. AI systems used for educational purposes must thus be validated to distinguish subtle variations across skin tones to prevent such diagnostic dilemmas.

Despite its promise, AI‐powered education tools present notable limitations. Chatbots might oversimplify or misinform if not supervised. Many models rely on incomplete datasets and lack regulation or clinical validation. Without transparency and oversight, these tools may inadvertently misguide rather than support patients. While many studies explore AI in diagnosis, this letter emphasizes its emerging role in personalized patient education, a crucial yet underrepresented facet. By demystifying treatments and improving engagement, AI can strengthen the therapeutic alliance between patients and physicians [6].

In conclusion, AI offers an unprecedented opportunity to revolutionize how we educate our patients in dermatology. It enables us to go beyond the one‐size‐fits‐all model and embrace customized, continuous, and interactive learning tailored to each individual. As we continue to integrate AI into our practices, let us ensure that patient education is not an afterthought, but a cornerstone of this technological transformation. Thoughtfully designed and ethically implemented, AI can be one of our most powerful allies in achieving not just better skin, but better‐informed and more empowered patients.

## Conflicts of Interest

The author declares no conflicts of interest.

## Data Availability

The data that supports the findings of this study are available in the cited references.
